# 2-(4-Chloro­anilino)quinoxaline

**DOI:** 10.1107/S1600536808038610

**Published:** 2008-11-26

**Authors:** Azila Idris, Wan Ainna Mardhiah Wan Saffiee, Zanariah Abdullah, Azahar Ariffin, Seik Weng Ng

**Affiliations:** a Department of Chemistry, University of Malaya, 50603 Kuala Lumpur, Malaysia

## Abstract

There are two mol­ecules in the asymmetric unit of the title compound, C_14_H_10_ClN_3_, with dihedral angles of 5.11 (10) and 13.61 (10)° between the aromatic ring systems. In the crystal structure, mol­ecules are linked by N—H⋯N hydrogen bonds, resulting in chains propagating in [010].

## Related literature

For the structure of 2-*N*-(4-chloro­anilino)pyridine, see: Fairuz *et al.* (2008 ▶).
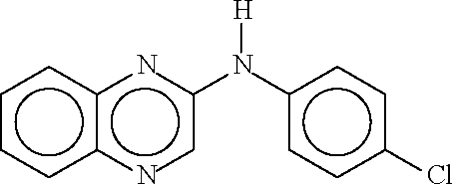


## Experimental

### 

#### Crystal data

C_14_H_10_ClN_3_
*M* *_r_* = 255.70
Orthorhombic, 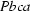

*a* = 12.155 (1) Å
*b* = 11.238 (1) Å
*c* = 35.421 (3) Å
*V* = 4838.3 (8) Å^3^
*Z* = 16
Mo *K*α radiation
μ = 0.30 mm^−1^
*T* = 100 (2) K
0.30 × 0.20 × 0.10 mm

#### Data collection

Bruker SMART APEX CCD diffractometer
Absorption correction: multi-scan (*SADABS*; Sheldrick, 1996 ▶) *T*_min_ = 0.916, *T*_max_ = 0.971
25622 measured reflections
5495 independent reflections
4111 reflections with *I* > 2σ(*I*)
*R*_int_ = 0.066

#### Refinement

*R*[*F* ^2^ > 2σ(*F* ^2^)] = 0.058
*wR*(*F* ^2^) = 0.135
*S* = 1.07
5495 reflections
331 parameters
2 restraints
H atoms treated by a mixture of independent and constrained refinement
Δρ_max_ = 0.31 e Å^−3^
Δρ_min_ = −0.28 e Å^−3^

### 

Data collection: *APEX2* (Bruker, 2007 ▶); cell refinement: *SAINT* (Bruker, 2007 ▶); data reduction: *SAINT*; program(s) used to solve structure: *SHELXS97* (Sheldrick, 2008 ▶); program(s) used to refine structure: *SHELXL97* (Sheldrick, 2008 ▶); molecular graphics: *X-SEED* (Barbour, 2001 ▶); software used to prepare material for publication: *publCIF* (Westrip, 2008 ▶).

## Supplementary Material

Crystal structure: contains datablocks global, I. DOI: 10.1107/S1600536808038610/hb2854sup1.cif

Structure factors: contains datablocks I. DOI: 10.1107/S1600536808038610/hb2854Isup2.hkl

Additional supplementary materials:  crystallographic information; 3D view; checkCIF report

## Figures and Tables

**Table 1:** Hydrogen-bond geometry (Å, °)

*D*—H⋯*A*	*D*—H	H⋯*A*	*D*⋯*A*	*D*—H⋯*A*
N1—H1⋯N6	0.88 (1)	2.24 (1)	3.086 (3)	160 (3)
N4—H4⋯N3^i^	0.88 (1)	2.19 (2)	3.010 (3)	155 (3)

Symmetry code: (i) 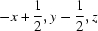.

**Fig. 1.**
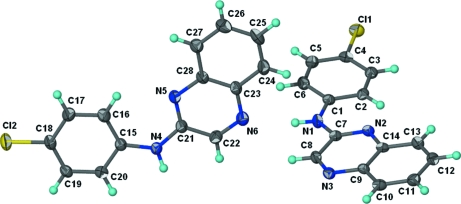
The molecular structure of (I) at the 70% probability level; hydrogen atoms are drawn as spheres of arbitrary radius.

## supplementary crystallographic information

### Comment

See the Abstract for details of the title compound, (I), (Fig. 1). See Table 1 for hydrogen bond information.  For a related structure, see: Fairuz *et al.* (2008).

### Experimental

Chloroquinoxaline (0.33 g, 0.2 mmol) and 4-chloroaniline (0.25 g, 0.2 mmol) were heated at 423–433 K for 5 h. The mixture was cooled and dissolved in water. The solution was extracted with chloroform. The chloroform extract was dried over sodium sulfate and the solvent evaporated. The product was recrystallized from chloroform to yield colourless prisms of (I).

### Refinement

The carbon-bound H-atoms were placed in calculated positions (C—H = 0.95 Å) and refined as riding with *U*(H) = 1.2*U*(C). The amino H-atoms were located in a difference Fourier map, and were refined with a distance restraint of N–H 0.88±0.01 Å.

### Crystal data

**Table:**

C_14_H_10_ClN_3_	*F*_000_ = 2112
*M**_r_* = 255.70	*D*_x_ = 1.404 Mg m^−^^3^
Orthorhombic, *P**b**c**a*	Mo *K*α radiation λ = 0.71073 Å
Hall symbol: -P 2ac 2ab	Cell parameters from 3986 reflections
*a* = 12.155 (1) Å	θ = 2.5–27.8º
*b* = 11.238 (1) Å	µ = 0.30 mm^−^^1^
*c* = 35.421 (3) Å	*T* = 100 (2) K
*V* = 4838.3 (8) Å^3^	Prism, colourless
*Z* = 16	0.30 × 0.20 × 0.10 mm

### Data collection

**Table:**

Bruker SMART APEX CCD diffractometer	5495 independent reflections
Radiation source: fine-focus sealed tube	4111 reflections with *I* > 2σ(*I*)
Monochromator: graphite	*R*_int_ = 0.066
*T* = 100(2) K	θ_max_ = 27.5º
ω scans	θ_min_ = 1.2º
Absorption correction: Multi-scan(SADABS; Sheldrick, 1996)	*h* = −15→15
*T*_min_ = 0.916, *T*_max_ = 0.971	*k* = −14→14
25622 measured reflections	*l* = −34→45

### Refinement

**Table:**

Refinement on *F*^2^	Secondary atom site location: difference Fourier map
Least-squares matrix: full	Hydrogen site location: inferred from neighbouring sites
*R*[*F*^2^ > 2σ(*F*^2^)] = 0.058	H atoms treated by a mixture of independent and constrained refinement
*wR*(*F*^2^) = 0.135	*w* = 1/[σ^2^(*F*_o_^2^) + (0.0485*P*)^2^ + 5.0859*P*] where *P* = (*F*_o_^2^ + 2*F*_c_^2^)/3
*S* = 1.07	(Δ/σ)_max_ = 0.001
5495 reflections	Δρ_max_ = 0.31 e Å^−^^3^
331 parameters	Δρ_min_ = −0.28 e Å^−^^3^
2 restraints	Extinction correction: none
Primary atom site location: structure-invariant direct methods	

### Fractional atomic coordinates and isotropic or equivalent isotropic displacement parameters (Å^2^)

**Table:**

	*x*	*y*	*z*	*U*_iso_*/*U*_eq_	
Cl1	0.71730 (5)	0.59065 (6)	0.569356 (19)	0.02492 (17)	
Cl2	0.02845 (6)	0.18094 (7)	0.22911 (2)	0.03264 (19)	
N1	0.51639 (18)	0.8613 (2)	0.44328 (6)	0.0193 (5)	
H1	0.481 (2)	0.813 (2)	0.4277 (7)	0.023*	
N2	0.59094 (17)	1.05427 (18)	0.44793 (6)	0.0167 (4)	
N3	0.45895 (17)	1.11409 (19)	0.38437 (6)	0.0176 (5)	
N4	0.22121 (17)	0.54504 (19)	0.33035 (6)	0.0192 (5)	
H4	0.1703 (18)	0.586 (2)	0.3421 (7)	0.023*	
N5	0.41121 (17)	0.5347 (2)	0.31927 (6)	0.0198 (5)	
N6	0.43633 (18)	0.7150 (2)	0.37487 (6)	0.0216 (5)	
C1	0.56706 (19)	0.8032 (2)	0.47383 (7)	0.0171 (5)	
C2	0.6235 (2)	0.8609 (2)	0.50277 (7)	0.0174 (5)	
H2	0.6304	0.9451	0.5026	0.021*	
C3	0.6698 (2)	0.7948 (2)	0.53204 (7)	0.0192 (5)	
H3	0.7084	0.8341	0.5518	0.023*	
C4	0.6597 (2)	0.6723 (2)	0.53243 (7)	0.0196 (5)	
C5	0.6043 (2)	0.6133 (2)	0.50399 (8)	0.0203 (6)	
H5	0.5977	0.5290	0.5045	0.024*	
C6	0.5582 (2)	0.6787 (2)	0.47463 (8)	0.0203 (5)	
H6	0.5204	0.6387	0.4549	0.024*	
C7	0.52621 (19)	0.9770 (2)	0.43137 (7)	0.0166 (5)	
C8	0.4600 (2)	1.0082 (2)	0.39899 (7)	0.0177 (5)	
H8	0.4152	0.9485	0.3879	0.021*	
C9	0.52583 (19)	1.1984 (2)	0.40114 (7)	0.0164 (5)	
C10	0.5304 (2)	1.3140 (2)	0.38633 (7)	0.0198 (5)	
H10	0.4862	1.3348	0.3652	0.024*	
C11	0.5988 (2)	1.3973 (2)	0.40237 (8)	0.0217 (6)	
H11	0.6026	1.4754	0.3922	0.026*	
C12	0.6633 (2)	1.3663 (2)	0.43405 (8)	0.0212 (6)	
H12	0.7101	1.4243	0.4451	0.025*	
C13	0.6594 (2)	1.2541 (2)	0.44907 (8)	0.0205 (6)	
H13	0.7028	1.2350	0.4705	0.025*	
C14	0.59083 (19)	1.1667 (2)	0.43268 (7)	0.0161 (5)	
C15	0.1823 (2)	0.4550 (2)	0.30593 (7)	0.0177 (5)	
C16	0.2491 (2)	0.3811 (2)	0.28402 (8)	0.0247 (6)	
H16	0.3268	0.3891	0.2850	0.030*	
C17	0.2014 (2)	0.2959 (3)	0.26090 (8)	0.0266 (6)	
H17	0.2466	0.2452	0.2461	0.032*	
C18	0.0884 (2)	0.2845 (2)	0.25939 (8)	0.0224 (6)	
C19	0.0212 (2)	0.3556 (2)	0.28118 (7)	0.0191 (5)	
H19	−0.0564	0.3466	0.2802	0.023*	
C20	0.0680 (2)	0.4399 (2)	0.30451 (7)	0.0189 (5)	
H20	0.0221	0.4884	0.3198	0.023*	
C21	0.3268 (2)	0.5814 (2)	0.33713 (7)	0.0183 (5)	
C22	0.3404 (2)	0.6720 (2)	0.36542 (8)	0.0211 (6)	
H22	0.2767	0.7019	0.3778	0.025*	
C23	0.5259 (2)	0.6708 (2)	0.35548 (7)	0.0187 (5)	
C24	0.6321 (2)	0.7168 (2)	0.36303 (8)	0.0249 (6)	
H24	0.6415	0.7777	0.3813	0.030*	
C25	0.7212 (2)	0.6729 (3)	0.34385 (8)	0.0286 (7)	
H25	0.7925	0.7037	0.3489	0.034*	
C26	0.7081 (2)	0.5829 (3)	0.31683 (8)	0.0288 (6)	
H26	0.7707	0.5532	0.3038	0.035*	
C27	0.6061 (2)	0.5372 (2)	0.30895 (8)	0.0236 (6)	
H27	0.5982	0.4760	0.2907	0.028*	
C28	0.5127 (2)	0.5815 (2)	0.32813 (7)	0.0193 (5)	

### Atomic displacement parameters (Å^2^)

**Table:**

	*U*^11^	*U*^22^	*U*^33^	*U*^12^	*U*^13^	*U*^23^
Cl1	0.0267 (3)	0.0231 (3)	0.0250 (4)	0.0045 (3)	−0.0042 (3)	0.0047 (3)
Cl2	0.0256 (4)	0.0359 (4)	0.0365 (4)	−0.0093 (3)	−0.0007 (3)	−0.0154 (3)
N1	0.0227 (11)	0.0162 (11)	0.0190 (12)	−0.0039 (9)	−0.0045 (9)	0.0001 (9)
N2	0.0150 (10)	0.0171 (11)	0.0180 (11)	0.0003 (8)	0.0001 (8)	0.0008 (9)
N3	0.0160 (10)	0.0170 (11)	0.0197 (11)	−0.0013 (8)	0.0004 (8)	0.0017 (9)
N4	0.0163 (11)	0.0187 (11)	0.0227 (12)	−0.0025 (9)	0.0027 (9)	−0.0045 (9)
N5	0.0187 (11)	0.0217 (11)	0.0192 (11)	−0.0023 (9)	0.0001 (9)	−0.0026 (9)
N6	0.0236 (11)	0.0203 (12)	0.0208 (12)	−0.0024 (9)	−0.0017 (9)	0.0006 (9)
C1	0.0134 (11)	0.0189 (13)	0.0191 (13)	0.0002 (10)	0.0018 (10)	0.0001 (10)
C2	0.0182 (12)	0.0167 (12)	0.0172 (13)	0.0003 (10)	0.0030 (10)	−0.0011 (10)
C3	0.0179 (12)	0.0215 (13)	0.0184 (13)	0.0011 (10)	0.0011 (10)	−0.0013 (11)
C4	0.0163 (12)	0.0221 (13)	0.0202 (14)	0.0031 (10)	0.0012 (10)	0.0029 (11)
C5	0.0189 (12)	0.0138 (12)	0.0283 (15)	−0.0016 (10)	0.0015 (11)	0.0027 (11)
C6	0.0198 (12)	0.0188 (13)	0.0224 (14)	−0.0029 (10)	−0.0019 (10)	0.0006 (11)
C7	0.0145 (11)	0.0172 (12)	0.0181 (13)	0.0014 (9)	0.0021 (10)	0.0007 (10)
C8	0.0157 (12)	0.0192 (13)	0.0181 (13)	−0.0031 (10)	0.0001 (10)	−0.0026 (10)
C9	0.0119 (11)	0.0192 (13)	0.0180 (13)	0.0007 (10)	0.0011 (9)	−0.0014 (10)
C10	0.0190 (12)	0.0200 (13)	0.0204 (14)	0.0037 (11)	−0.0002 (10)	0.0041 (11)
C11	0.0216 (13)	0.0152 (13)	0.0284 (15)	0.0007 (10)	0.0001 (11)	0.0013 (11)
C12	0.0181 (12)	0.0192 (13)	0.0263 (15)	−0.0015 (10)	−0.0014 (11)	−0.0012 (11)
C13	0.0186 (13)	0.0215 (13)	0.0215 (14)	−0.0013 (10)	−0.0015 (10)	−0.0002 (11)
C14	0.0127 (11)	0.0178 (12)	0.0179 (13)	0.0028 (9)	0.0022 (9)	0.0018 (10)
C15	0.0184 (12)	0.0172 (12)	0.0177 (13)	−0.0017 (10)	−0.0008 (10)	0.0009 (10)
C16	0.0170 (13)	0.0268 (15)	0.0305 (16)	−0.0045 (11)	0.0038 (11)	−0.0055 (12)
C17	0.0215 (14)	0.0270 (15)	0.0312 (16)	−0.0006 (11)	0.0026 (12)	−0.0096 (12)
C18	0.0215 (13)	0.0213 (14)	0.0245 (15)	−0.0053 (10)	−0.0031 (11)	0.0002 (11)
C19	0.0151 (12)	0.0198 (13)	0.0223 (14)	−0.0033 (10)	−0.0013 (10)	0.0033 (11)
C20	0.0185 (12)	0.0205 (13)	0.0178 (13)	0.0006 (10)	0.0013 (10)	0.0023 (10)
C21	0.0195 (12)	0.0147 (12)	0.0207 (14)	−0.0036 (10)	−0.0013 (10)	0.0025 (10)
C22	0.0234 (13)	0.0169 (13)	0.0230 (14)	−0.0003 (11)	0.0011 (11)	−0.0003 (11)
C23	0.0210 (12)	0.0157 (12)	0.0195 (13)	−0.0031 (10)	−0.0032 (10)	0.0028 (10)
C24	0.0279 (15)	0.0190 (14)	0.0278 (16)	−0.0048 (11)	−0.0061 (12)	−0.0003 (12)
C25	0.0199 (13)	0.0343 (16)	0.0316 (16)	−0.0071 (12)	−0.0070 (11)	0.0028 (13)
C26	0.0200 (13)	0.0350 (16)	0.0314 (16)	−0.0005 (12)	0.0002 (12)	0.0007 (13)
C27	0.0213 (13)	0.0250 (14)	0.0244 (15)	−0.0018 (11)	0.0003 (11)	−0.0023 (12)
C28	0.0181 (12)	0.0194 (13)	0.0203 (14)	−0.0018 (10)	−0.0022 (10)	0.0026 (11)

### Geometric parameters (Å, °)

**Table:**

Cl1—C4	1.745 (3)	C10—C11	1.375 (4)
Cl2—C18	1.743 (3)	C10—H10	0.9500
N1—C7	1.373 (3)	C11—C12	1.412 (4)
N1—C1	1.406 (3)	C11—H11	0.9500
N1—H1	0.883 (10)	C12—C13	1.369 (4)
N2—C7	1.310 (3)	C12—H12	0.9500
N2—C14	1.374 (3)	C13—C14	1.413 (3)
N3—C8	1.297 (3)	C13—H13	0.9500
N3—C9	1.383 (3)	C15—C16	1.397 (4)
N4—C21	1.368 (3)	C15—C20	1.400 (3)
N4—C15	1.412 (3)	C16—C17	1.387 (4)
N4—H4	0.877 (10)	C16—H16	0.9500
N5—C21	1.314 (3)	C17—C18	1.381 (4)
N5—C28	1.377 (3)	C17—H17	0.9500
N6—C22	1.306 (3)	C18—C19	1.379 (4)
N6—C23	1.380 (3)	C19—C20	1.380 (4)
C1—C2	1.394 (4)	C19—H19	0.9500
C1—C6	1.403 (4)	C20—H20	0.9500
C2—C3	1.394 (4)	C21—C22	1.438 (4)
C2—H2	0.9500	C22—H22	0.9500
C3—C4	1.382 (4)	C23—C28	1.404 (4)
C3—H3	0.9500	C23—C24	1.415 (4)
C4—C5	1.382 (4)	C24—C25	1.371 (4)
C5—C6	1.391 (4)	C24—H24	0.9500
C5—H5	0.9500	C25—C26	1.401 (4)
C6—H6	0.9500	C25—H25	0.9500
C7—C8	1.444 (3)	C26—C27	1.370 (4)
C8—H8	0.9500	C26—H26	0.9500
C9—C10	1.403 (4)	C27—C28	1.413 (4)
C9—C14	1.414 (3)	C27—H27	0.9500
			
C7—N1—C1	129.7 (2)	C12—C13—H13	120.0
C7—N1—H1	115.4 (19)	C14—C13—H13	120.0
C1—N1—H1	114.2 (19)	N2—C14—C9	122.9 (2)
C7—N2—C14	115.6 (2)	N2—C14—C13	118.5 (2)
C8—N3—C9	116.8 (2)	C9—C14—C13	118.6 (2)
C21—N4—C15	129.5 (2)	C16—C15—C20	119.0 (2)
C21—N4—H4	115 (2)	C16—C15—N4	124.9 (2)
C15—N4—H4	116 (2)	C20—C15—N4	116.2 (2)
C21—N5—C28	115.9 (2)	C17—C16—C15	119.7 (2)
C22—N6—C23	116.4 (2)	C17—C16—H16	120.1
C2—C1—C6	119.1 (2)	C15—C16—H16	120.1
C2—C1—N1	124.4 (2)	C18—C17—C16	120.2 (3)
C6—C1—N1	116.4 (2)	C18—C17—H17	119.9
C1—C2—C3	119.8 (2)	C16—C17—H17	119.9
C1—C2—H2	120.1	C19—C18—C17	120.9 (3)
C3—C2—H2	120.1	C19—C18—Cl2	118.9 (2)
C4—C3—C2	120.2 (2)	C17—C18—Cl2	120.1 (2)
C4—C3—H3	119.9	C18—C19—C20	119.3 (2)
C2—C3—H3	119.9	C18—C19—H19	120.4
C3—C4—C5	121.0 (2)	C20—C19—H19	120.4
C3—C4—Cl1	119.7 (2)	C19—C20—C15	120.9 (2)
C5—C4—Cl1	119.3 (2)	C19—C20—H20	119.5
C4—C5—C6	119.1 (2)	C15—C20—H20	119.5
C4—C5—H5	120.4	N5—C21—N4	121.9 (2)
C6—C5—H5	120.4	N5—C21—C22	121.9 (2)
C5—C6—C1	120.8 (2)	N4—C21—C22	116.2 (2)
C5—C6—H6	119.6	N6—C22—C21	122.9 (2)
C1—C6—H6	119.6	N6—C22—H22	118.6
N2—C7—N1	122.9 (2)	C21—C22—H22	118.6
N2—C7—C8	122.0 (2)	N6—C23—C28	120.7 (2)
N1—C7—C8	115.2 (2)	N6—C23—C24	119.6 (2)
N3—C8—C7	123.0 (2)	C28—C23—C24	119.7 (2)
N3—C8—H8	118.5	C25—C24—C23	119.7 (3)
C7—C8—H8	118.5	C25—C24—H24	120.1
N3—C9—C10	119.8 (2)	C23—C24—H24	120.1
N3—C9—C14	119.7 (2)	C24—C25—C26	120.5 (3)
C10—C9—C14	120.5 (2)	C24—C25—H25	119.7
C11—C10—C9	120.0 (2)	C26—C25—H25	119.7
C11—C10—H10	120.0	C27—C26—C25	120.8 (3)
C9—C10—H10	120.0	C27—C26—H26	119.6
C10—C11—C12	119.7 (2)	C25—C26—H26	119.6
C10—C11—H11	120.1	C26—C27—C28	119.8 (3)
C12—C11—H11	120.1	C26—C27—H27	120.1
C13—C12—C11	121.1 (2)	C28—C27—H27	120.1
C13—C12—H12	119.4	N5—C28—C23	122.2 (2)
C11—C12—H12	119.4	N5—C28—C27	118.4 (2)
C12—C13—C14	120.0 (2)	C23—C28—C27	119.4 (2)
			
C7—N1—C1—C2	12.7 (4)	C21—N4—C15—C16	2.0 (4)
C7—N1—C1—C6	−167.7 (3)	C21—N4—C15—C20	−178.8 (3)
C6—C1—C2—C3	−0.3 (4)	C20—C15—C16—C17	1.1 (4)
N1—C1—C2—C3	179.3 (2)	N4—C15—C16—C17	−179.7 (3)
C1—C2—C3—C4	−0.1 (4)	C15—C16—C17—C18	0.3 (4)
C2—C3—C4—C5	0.2 (4)	C16—C17—C18—C19	−1.2 (4)
C2—C3—C4—Cl1	−179.64 (19)	C16—C17—C18—Cl2	177.8 (2)
C3—C4—C5—C6	0.0 (4)	C17—C18—C19—C20	0.6 (4)
Cl1—C4—C5—C6	179.86 (19)	Cl2—C18—C19—C20	−178.3 (2)
C4—C5—C6—C1	−0.4 (4)	C18—C19—C20—C15	0.8 (4)
C2—C1—C6—C5	0.5 (4)	C16—C15—C20—C19	−1.6 (4)
N1—C1—C6—C5	−179.1 (2)	N4—C15—C20—C19	179.1 (2)
C14—N2—C7—N1	−179.6 (2)	C28—N5—C21—N4	178.5 (2)
C14—N2—C7—C8	0.6 (3)	C28—N5—C21—C22	−2.7 (4)
C1—N1—C7—N2	1.3 (4)	C15—N4—C21—N5	2.2 (4)
C1—N1—C7—C8	−178.8 (2)	C15—N4—C21—C22	−176.7 (2)
C9—N3—C8—C7	0.5 (4)	C23—N6—C22—C21	1.6 (4)
N2—C7—C8—N3	−0.8 (4)	N5—C21—C22—N6	1.0 (4)
N1—C7—C8—N3	179.4 (2)	N4—C21—C22—N6	179.8 (2)
C8—N3—C9—C10	178.7 (2)	C22—N6—C23—C28	−2.2 (4)
C8—N3—C9—C14	0.0 (3)	C22—N6—C23—C24	177.3 (2)
N3—C9—C10—C11	−178.5 (2)	N6—C23—C24—C25	179.9 (3)
C14—C9—C10—C11	0.3 (4)	C28—C23—C24—C25	−0.6 (4)
C9—C10—C11—C12	−0.8 (4)	C23—C24—C25—C26	0.0 (4)
C10—C11—C12—C13	0.3 (4)	C24—C25—C26—C27	0.2 (4)
C11—C12—C13—C14	0.6 (4)	C25—C26—C27—C28	0.3 (4)
C7—N2—C14—C9	−0.2 (3)	C21—N5—C28—C23	2.1 (4)
C7—N2—C14—C13	−179.6 (2)	C21—N5—C28—C27	−178.1 (2)
N3—C9—C14—N2	−0.1 (4)	N6—C23—C28—N5	0.4 (4)
C10—C9—C14—N2	−178.9 (2)	C24—C23—C28—N5	−179.1 (2)
N3—C9—C14—C13	179.3 (2)	N6—C23—C28—C27	−179.4 (2)
C10—C9—C14—C13	0.6 (4)	C24—C23—C28—C27	1.1 (4)
C12—C13—C14—N2	178.5 (2)	C26—C27—C28—N5	179.3 (3)
C12—C13—C14—C9	−1.0 (4)	C26—C27—C28—C23	−0.9 (4)

### Hydrogen-bond geometry (Å, °)

**Table:**

*D*—H···*A*	*D*—H	H···*A*	*D*···*A*	*D*—H···*A*
N1—H1···N6	0.88 (1)	2.24 (1)	3.086 (3)	160 (3)
N4—H4···N3^i^	0.88 (1)	2.19 (2)	3.010 (3)	155 (3)

Symmetry codes: (i) −*x*+1/2, *y*−1/2, *z*.
